# Cell–cell communication in bacteria: new insights and future trends

**DOI:** 10.1099/mic.0.001736

**Published:** 2026-07-30

**Authors:** Vittorio Venturi, Livia Leoni

**Affiliations:** 1 African Genome Center, University Mohammed VI Polytechnic (UM6P), Ben Guerir, Morocco; 2 International Centre for Genetic Engineering and Biotechnology (ICGEB), Trieste, Italy; 3 Department of Science, University Roma Tre, Rome, Italy

**Keywords:** quorum sensing, bacteria, CCCB 2025 conference

In October 2025, the International Centre for Genetic Engineering and Biotechnology (ICGEB) hosted the Arturo Falaschi Conference entitled ‘Cell–cell communication in bacteria: new insights and future trends’ in Trieste, Italy. The meeting brought together established leaders and emerging researchers from across the globe to discuss recent advances and evolving concepts in microbial signalling.

The oral presentations reflected both the historical foundations of the field and its continued expansion into new types and dimensions of cell-cell signalling in micro-organisms. While research on classical quorum sensing (QS) systems remains central, with *Pseudomonas aeruginosa* continuing to serve as the undisputed model organism, the conference highlighted how the original QS paradigm is being revisited and reshaped in the context of complex microbial communities, host-associated environments and spatially structured populations.

Importantly, several contributions highlighted the emergence of novel modes of cell–cell signalling in previously underexplored micro-organisms. These included communication systems in archaea, signalling mechanisms in marine bacteria, phytosphere-associated microbes and interkingdom signalling processes linking bacteria with plants, animals and even microalgae.

## Revisiting the foundations of QS

The conference opened with Peter Greenberg revisiting the foundations of acyl-homoserine lactone (AHL) QS, reminding us how far the field has progressed since its discovery and how many fundamental questions remain. George Salmond showed how these signals in Gram-negative enterobacteria coordinate plant pathogenesis, antibiotic production and gas vesicle formation, integrating multiple functions in a cell-density- and environment-dependent manner. While the molecular details of AHL signal synthesis and receptor activation are now well understood, predicting signalling chemistry and LuxR-family/AHL specificity and deciphering communication in polymicrobial environments remain major challenges.

The theme of moving from simplified laboratory models to ecological complexity resonated throughout the meeting. For example, Jon Paczkowski and colleagues highlighted the dynamic nature of QS in *P. aeruginosa*, emphasizing how signalling networks are rewired during infection. Similarly, work presented by Morgana Letizia demonstrated that QS-controlled genes behave very differently in chronic human infections compared with pure laboratory cultures, stressing the importance of studying bacterial signalling in clinically relevant contexts. Beyond chemical and diffusible signalling, Sigal Ben-Yehuda’s work on bacterial nanotubes provided a striking real-time visualization of contact-dependent communication, revealing a structural and physical layer of cell–cell interaction.

## LuxR solos and signal plasticity

Several talks challenged the classical LuxI/LuxR QS paradigm. Cristina Bez and colleagues presented evidence that LuxR solos, i.e. QS LuxRs devoid of a cognate LuxI, are extremely widespread in natural microbiomes and often associated with previously uncharacterized biosynthetic gene clusters. It is striking that, more than four decades after the discovery of AHLs in the early 1980s, we have deciphered only fragments of the vast chemical language microbes use not only to communicate with one another but also across kingdoms. An interesting example comes from the work of Ralf Heermann on *Photorhabdus luminescens*, which provided evidence that LuxR solos can regulate the switch between pathogenic and mutualistic lifestyles through the detection of host-derived signalling molecules.

Studies presented by the groups of Eric Déziel and Martin Welch further illustrated the plasticity of QS networks, showing how mutations in signal synthesis or receptor genes can drive rewiring of QS regulation. These studies provide insight into alternative virulence pathways and could offer a foundation for better understanding and combating chronic *P. aeruginosa* infections, especially in cystic fibrosis.

Overall, the evidence suggests that QS is not a rigid hierarchy but a flexible regulatory cascade capable of remarkable adaptation under selective pressure.

## QS spatial structure and multicellular organization

A recurring theme was the importance of spatial and lineage effects in shaping communication outcomes. Xiaohe Liu and colleagues demonstrated that QS activation within microcolonies is strongly influenced by cell lineage rather than simple diffusion-driven proximity. Complementing this, Marvin Whiteley’s group used spatially resolved single-cell RNA sequencing to show how localized interspecies interactions generate transcriptional heterogeneity in biofilms. Matthew Parsek revisited the relationship between QS and biofilm matrix production. Steve Diggle showed how spatial structuring at the micron scale plays a crucial role in maintaining cooperation by restricting cheater access to public goods. Together, these presentations focused on how signalling networks intersect with multicellular organization and antimicrobial tolerance.

## Emergent cell–cell communication systems

Even though bacteria, and in particular *P. aeruginosa*, remain unquestionably the major models for QS studies, it was encouraging to see that new non-bacterial models for studying QS are increasingly emerging. Mecky Pohlschroder introduced a tractable archaeal QS system in *Haloferax volcanii*, revealing conserved principles and domain-specific innovations. Georg Pohnert described femtomolar pheromone signalling in diatom reproduction, demonstrating that chemical communication in microbial systems extends deep into the eukaryotic world.

## signalling in host–microbe and polymicrobial contexts

Host-derived signals and interkingdom interactions were prominently featured. Liang Yang demonstrated that RetS in *P. aeruginosa* functions as a receptor for human-derived molecules, linking bacterial decision-making directly to host metabolites. Laurence Rahme, one of the pioneers in the field of QS based on alkylquinolones, connected QS to host epigenetic reprogramming via lactylation, revealing a previously underappreciated layer of host–pathogen interplay. Studies on polymicrobial competition by Rolf Kümmerli, Ajai Dandekar and Tim Miyashiro showed that bacteria integrate QS with secretion systems and metabolic strategies to discriminate between competitors and environmental cues. These works collectively also point toward a system-level understanding of real ecosystems.

## Blocking communication: from concept to chemistry

Finally, anti-virulence approaches targeting signalling and regulatory pathways remain a very active area of research in cell–cell communication. Several posters and oral presentations addressed this topic. Julia Van Kessel and Helen Blackwell showcased sophisticated chemical strategies to destabilize QS regulators and modulate signalling networks. These studies highlight a continuous interest in the translational dimension of the field, where mechanistic insight is critical for the rational design of anti-QS strategies. The final lecture was given by Miguel Camara, who highlighted some of the successes, frustrations and future directions in this evolving field. Despite the large number of studies on QS inhibitors and their promising *in vitro* results, their translation into clinical applications still faces several challenges, including the complexity of QS regulatory networks, the limited availability of suitable animal models and the need for further evidence regarding efficacy and safety. Consequently, QS inhibitors are now considered promising therapeutic candidates, but additional studies are required to establish their efficacy, safety and feasibility for clinical use. Importantly, as also highlighted during the discussion following Julia Van Kessel’s presentation, some of the first successful real-world applications of QS inhibitors may emerge in zootechnical settings such as aquaculture, where translational barriers are comparatively lower than in human clinical contexts and where experimental trials have reported very promising results.

## Emerging directions from the poster sessions

The poster sessions evidenced the vitality and diversity of the field. With nearly 50 poster presentations representing institutions across Europe, Africa, North America, the Middle East, Asia and beyond, the breadth of participation reflected the internationality of microbial communication research. The abstracts revealed several recurring analytical trends: the expanding focus on LuxR solos and non-canonical signalling modules; the integration of metabolomics, transcriptomics and chemical biology to uncover previously hidden signalling layers; and increasing attention to interspecies and interkingdom communication in natural and host-associated microbiomes. A significant proportion of contributions addressed clinically relevant pathogens such as *P. aeruginosa*, *Vibrio* spp. and *S. aureus*, often through mechanistic or anti-virulence topics. Posters also highlighted communication in plant-associated systems, marine environments, engineered gut models and extreme habitats, reflecting a shift towards ecological realism. Collectively, the poster presentations illustrated a field that is not only expanding in conceptual scope but also diversifying methodologically and geographically, with strong engagement from early-career researchers.

## Honouring the pioneers while looking towards the future

Across the sessions, a recurring message emerged: microbial communication is no longer viewed simply as a density-sensing mechanism, but as a highly adaptable, evolutionarily modular and environmentally responsive regulatory strategy for community behaviour. The field is increasingly embracing spatial structure, metabolic integration, interkingdom dialogue, new classes of signals and the reality of complex communities in natural environments. This recurrent conference will also need to embrace increasingly diverse types of cell–cell signalling systems and highlight their relevance in natural environments.

This conference also marked an important generational moment for the field. Several scientists whose work has profoundly shaped our understanding of bacterial communication are now entering retirement. George Salmond’s pioneering research on QS, antibiotic production, morphogenesis and regulatory networks in enterobacteria helped define the molecular logic linking cell–cell signalling to virulence and ecological adaptation. His work consistently bridged regulatory biology with evolutionary and applied perspectives, influencing both fundamental microbiology and translational thinking. Miguel Cámara has been central to advancing the mechanistic and therapeutic dimensions of QS research. His contributions to understanding signal perception, regulatory hierarchies in *P. aeruginosa* and anti-virulence strategies have been instrumental in shaping approaches to targeting bacterial communication. Paul Williams, although unable to attend this meeting, has made impactful contributions to the study of QS, particularly in *P. aeruginosa*, and to the development of QS inhibitors as anti-virulence agents, helping to bridge fundamental discovery with clinical application. Pete Greenberg’s foundational work on AHL-mediated QS established the conceptual and molecular framework upon which the field is built, from LuxI/LuxR regulatory circuits to the social and evolutionary dimensions of bacterial collective behaviour. Importantly, some of these scientists are contributing a dedicated historical perspective to this Special Collection, reflecting on the development and current trends of the field they helped shape. It is fitting that a conference focused on ‘new insights and future trends’ also acknowledges the intellectual foundations laid by these pioneers. This Special Collection of the CCCB conference of 2025, spanning mechanistic insight, ecological complexity, host interaction and therapeutic innovation, is deeply rooted in their contributions.

## Abbreviations

AHL: acyl-homoserine lactone
QS: quorum sensing

